# Early or delayed umbilical cord clamping? Experiences and perceptions of nurse-midwives and obstetricians at a regional referral hospital in Tanzania

**DOI:** 10.1371/journal.pone.0234854

**Published:** 2020-06-22

**Authors:** Dorkasi Lushindiho Mwakawanga, Lilian Teddy Mselle

**Affiliations:** 1 Department of Community Health Nursing, School of Nursing, Muhimbili University of Health and Allied Sciences, Dar es Salaam, Tanzania; 2 Department of Clinical Nursing, School of Nursing, Muhimbili University of Health and Allied Sciences, Dar es Salaam, Tanzania; University of Brighton, UNITED KINGDOM

## Abstract

**Background:**

Umbilical cord clamping is a crucial step during the third stage of labour that separates the newborn from the placenta. Despite the available evidence that delayed umbilical cord clamping is more beneficial to infants, as well as the existence of 2014 WHO recommendation that the umbilical cord should be clamped between 1 and 3 minutes, its implementation is still low in many countries including Tanzania.

**Objective:**

This study describes the experiences and perceptions of nurse-midwives`and obstetricians`about the timing of umbilical cord clamping at a regional referral hospital in Tanzania.

**Methods:**

A descriptive qualitative study design that adopted a purposeful sampling strategy to recruit 19 participants was used. Nine semi-structured interviews with six nurse-midwives`and three obstetricians`, as well as one focus group discussion with ten nurse-midwives`were conducted. Thematic analysis guided the analysis of data.

**Results:**

Three main themes generated from the data, each having 2 to 5 subthemes. 1. Experiences about the timing of umbilical cord clamping. 2. Perceptions about the umbilical cord clamping. 3. Factors influencing the practice of delayed umbilical cord clamping to improve newborn health outcomes.

**Conclusion:**

Although the nurse-midwives`and obstetricians`commonly practiced clamping the umbilical cord immediately after delivery, they understood that delayed cord clamping has a potential benefit of oxygenation to the newborn in the event of the need for resuscitation. To move forward with the good practice in maternal and newborn care, proper pre-service and providers training on matters underlying childbirth is essential to address the gap of knowledge. Delayed cord clamping should be practiced widely to improve the health outcomes of the newborn.

## Introduction

Umbilical cord clamping is done to separate the newborn from the placenta and is a crucial step during the third stage of labour. The timing of cord clamping is still a controversial issue worldwide. The World Health Organisation (WHO) defines early cord clamping as the clamping of the umbilical cord within the first 60 seconds of birth. Delayed cord clamping is defined as the clamping of the cord within 1 to 3 minutes of birth, or when the umbilical cord pulsations have stopped [1, 2]. The most recent WHO Guideline on delayed cord clamping (2014) recommends that, even when positive pressure ventilation is required, the cord should not be clamped earlier than 60 seconds in both term and preterm babies [1]. This is a change from the WHO Basic Newborn Resuscitation Guideline of 2012 which emphasized that early cord clamping is recommended when the neonate is asphyxiated and needs to be moved immediately for resuscitation [3, 4], and is otherwise contraindicated. Delayed cord clamping combined with resuscitation found to be a suitable option for asphyxiated new-borns than immediate cord clamping [5, 6].

Delayed cord clamping (DCC) has reported having better neonatal outcomes and appears to be safe, feasible, and effective with no adverse effects in both term and preterm new-borns [7]. Improvement in haematocrit levels and reduction in hospital mortality being the most reported significant effects associated with delayed cord clamping after the initiation of ventilation [8, 9]. Moreover, studies have reported DCC to be associated with a decreased risk of iron deficiency anaemia (due to an increase in haemoglobin and haematocrit levels), a decreased rate of intravascular haemorrhage, less surfactant, an increase in cerebral oxygenation, mechanical ventilation and enhanced cardiovascular stability by increasing pulmonary blood flow to prevent ischemic conditions [1, 7, 10–12]. These deficiencies often require blood transfusions in the neonate, and the fact that there is substantial improvement in these parameters has also been associated with improved neurodevelopment outcomes up to 4 years of age [13–16].

Early cord clamping (ECC) may be associated with negative neonatal outcomes such as hypoxia, infections, delayed psychomotor development, and anaemia [17, 18]. In 2015, Tanzania’s Ministry of Health, Community Development, Gender, Elderly and Children (MoHCDGEC) reported that 43 infants out of every 1,000 live births die within the first year of life and neonatal deaths totalled 25 for every 1,000 live births. Most of the neonatal deaths were caused by birth asphyxia. In addition, 58% of children under five had anaemia in 2015 [19]. It has been shown that children with iron deficiency anaemia have an increased risk of impaired neurodevelopment, potentially affecting their cognitive, motor, and behavioural abilities [17]. The WHO recommendations on delayed umbilical cord clamping may reduce the risk of anaemia and hypoxia to infants, and may also reduce the risk of postpartum haemorrhage to the mother [1] because it gives the midwife or obstetrician time to actively manage the third stage of labour. Nurse-midwives and obstetricians are the key personnel in the conduction of deliveries, and their practice of cord clamping has great potential to improve neonatal health outcomes. However, studies have reported that the delayed cord clamping recommendation is less practiced than it should be [20, 21]. This appears to be due to the lack of policies and guidelines regarding the timing of umbilical cord clamping [21–24].

In Tanzania Maternity care that is health services provided to women, babies, and families throughout the whole pregnancy, during labour and birth, and after birth for up to six weeks, is provided at all levels of health facilities from primary to tertiary health care facilities. Nurse-midwives and obstetricians are responsible for the provision of maternity care including the provision of clean and safe delivery. High quality maternal and newborn care during pregnancy, birth, and postpartum require effective interventions [25]. Delayed cord clamping is one among the recommended interventions for improved newborn nutrition outcomes, that requires skills and a positive attitude of health care providers [25]. National health policy has prioritized maternity care thus ensuring that all women during pregnancy, birth and postpartum get access to quality and safe care in the health care facilities [26]. The Tanzania and Demographic Health Survey of 2016 has reported that 98% of pregnant women attended ANC services at least once, however, only 63% of these gave birth in health facility of which 64% were assisted by a skilled birth attendant [19]. Women are reported to be reluctant to utilize the health facility during childbirth due to the health care provider's negative attitude they experience when they encounter them [27–29].

To date, studies on umbilical cord clamping practices in Tanzania have focused on the neonatal outcomes following cord clamping after the onset of spontaneous respirations, and on its benefits to cardiovascular stability [30, 31]. Little is known regarding the nurse-midwives`and obstetricians’ experiences and perceptions regarding the timing of umbilical cord clamping. This study aimed at describing the experiences and perceptions of nurse-midwives`and obstetricians`regarding the timing of umbilical cord clamping at a regional referral hospital in Dar es Salaam. Furthermore, it described the factors which may influence the application of evidence into practice.

## Materials and methods

### Study design and setting

A descriptive qualitative research design [32] was used to explore nurse-midwives`and obstetricians’ experiences and perceptions regarding the timing of umbilical cord clamping. The study was conducted at a regional referral hospital in Dar es Salaam. This is also a secondary level referral hospital for both complicated and uncomplicated deliveries. The majority of women residing in Dar es Salaam come here for low-risk deliveries, as well as both in and outpatient health care services. It has an average of 1000 deliveries per month. In 2017, the hospital had 16 maternal deaths and 390 neonatal deaths. Of the neonatal deaths, 262 were caused by birth asphyxia [33].

### Participants’ recruitment and data collection

This study included 19 participants who were purposefully recruited [34] for the study. These were 6 nurse-midwives`and 3 obstetricians`who were interviewed and 10 nurse-midwives`who participated in the discussion. However, the qualitative sample size has no rule it depends on what the researcher wants to know, the purpose of the research study, and what can be done with available time and resources. It is further recommended that the minimum samples for qualitative research should be based on expected reasonable coverage of the phenomenon given the purpose of the study and interest [35]. The nurse in charge of the labour ward was asked to identify nurse-midwives`and obstetricians`who had worked in the labour ward for more than one year. The researcher (a midwife) approached the identified midwives`and obstetricians`and administered a short screening questionnaire to check if they meet the inclusion criteria. Those who met the criteria were asked to participate in the study after they were explained about the purpose of the study, issues of confidentiality, and the voluntary nature of their participation.

Data were collected using semi-structured interviews and focus group discussion. An audio recorder was used during both the interviews as well as the discussion to ensure that all narratives from the participants are captured. The participants provided written consent for the conversations to be recorded.

### Semi-Structured Interviews (SSI)

Nine semi-structured interviews were conducted in the Kiswahili language, the native language spoken by the participants and authors. Interviews were carried out at the hospital in a comfortable, private room that was quiet with no distractions. The semi-structured interview guide used (see Table 1) contained open-ended and non-directive questions to allow in-depth information from participants. Interviews were conducted until saturation occurred, when there was no new information obtained from the participants, and redundancy was achieved [32]. All interview sessions took not more than 40 minutes.

**Table 1:** Interview guide for nurse-midwives` and obstetricians`.

Interview questions and probes	
1	Could you please tell me about your experience regarding the timing of umbilical cord clamping after the delivery of the baby? what do you consider before you clamp the cord? how long do you wait until you clamp the cord? why so?
2	What issues that would make you decide the time of cord clamping? what are the reasons for immediate cord clamping? delayed cord clamping?
	**Probes**; benefits of ECC, benefits DCC, risks or disadvantages of ECC, risks or disadvantages of DCC
3	What are the factors influencing the timing of umbilical cord clamping?
	**Probe;** what are the personal factors? what are the health facility factors?
4	What do you think about the timing of UCC? **Probe;** what do you suggest?
5	In your opinion, what would you suggest for the nurse-midwives and obstetricians to practice based on evidence?

### Focus group discussion

Focus group discussion (FGD) with 10 nurse-midwives`was also conducted. The focus group discussion was conducted with only nurse-midwives`because of the limited number of obstetricians`in this regional referral hospital, it was not possible to have an adequate number of this category of participants for the focus group discussion [34]. The discussion was moderated by the first author using the FGD guide. Early in the discussion, the purpose of the study was explained and the participants were encouraged to actively participate in the discussion. The trained research assistant recorded the participant’s non-verbal communications during the discussion that supplemented the audio recorded information. The discussion took about 45 minutes.

### Data analysis

Braun and Clarke`s thematic analysis guided the analysis of data [36, 37]. The thematic analysis provides a flexible method of data analysis and allows researchers with various methodological backgrounds to participate [38]. The thematic analytical approach involved six main steps. Audio-recorded data from the interviews and discussion were transcribed verbatim by the researcher and were read and re-read to become familiar with the data to get a general understanding of the participants’ accounts. A list of potential and initial codes reflected the pre-determined themes that were created through data reduction. Sub-themes and themes were then generated from the general list of codes created (see Table 2). The focus was on broad patterns in the data and the coded data were combined based on their relationships to form the themes. Themes were reviewed and discussed by the researchers, discrepancies were noted and identified themes were finalized.

**Table 2:** Summary of findings showing themes and subthemes.

S/N	Themes	Sub-Themes
1	Experiences about the timing of umbilical cord clamping and cutting	• Clamping the umbilical cord within 60 seconds • Clamping the umbilical cord after cessation of cord pulsations • Timing of the umbilical cord clamping is determined by the condition of the baby
2	Perceptions about the umbilical cord clamping and cutting	• Perceived benefits of DCC • Perceived risks of DCC
3	Factors influencing the practice of delayed cord clamping and cutting	• Good knowledge about umbilical cord clamping • Using guidelines and Standard Operating Procedures • Adequate resources • Supportive supervision • Availability of on-the-job training

### Ethical consideration

The ethical approval to conduct this study was obtained from Muhimbili University of Health and Allied Sciences (MUHAS), Senate Research and Publications Committee (Ref. no. DA.287/298/01A/). Medical officer-in-charge of the hospital granted permission for data collection. Written informed consent was sought from participants after they were fully informed about the purpose of the study and the use of audio recorder during the interviews and discussion. Participants were also informed about the right to withdraw and they were assured that their participation was voluntary. Confidentiality and anonymity were maintained by assigning numbers to participants during data collection instead of names.

## Results

### Socio-demographic characteristics of the participants

A total of 19 participants were involved in this study. The study was comprised of two groups of participants; sixteen (16) nurse-midwives`and three (3) obstetricians`. Among the nurse-midwives, 6 were interviewed and 10 were involved in focus group discussion. Eight (8) had a certificate, seven (7) had a diploma and one (1) had a degree in nursing. Among all participants 14 were females and 8 had worked in the labour ward for more than 5 years. Table 3 shows the summary of the demographic profile of the participants from semi-structured interviews and focus group discussion.

**Table 3:** Socio-demographic characteristics of SSI and FGD participants.

Participant`s characteristics	Frequency (n = 19)
**Age (years)**	
<35	9
Above 35	10
**Sex**	
Female	14
Male	5
**Profession**	
Nurse-midwives	16
Obstetricians	3
**Profession education level**	
Certificate in Nursing	8
Diploma in nursing	7
Degree in Nursing	1
Master’s degree in obstetrics	3
**Years of training in the health field**	
2 years	8
3–4 years	8
Above 5 years	3
**Years of working experience**	
1–2	4
3–4	7
5 and above	8

#### Theme one: Experiences about the timing of umbilical cord clamping and cutting

This theme illustrates about experiences of participants regarding the timing of umbilical cord clamping after the delivery of the baby. It presents information about participants`considerations before they clamp and cut the umbilical cord after the delivery of the baby, specifically how long it takes to clamp and cut the cord.

#### Sub-theme 1: Clamping the umbilical cord within 60 seconds

Participants in this study reported their experiences of clamping the umbilical cord immediately when the baby is born and after the baby’s cry or breath spontaneously.

*“(*. . .*) when the baby is out*, *I placed on the mother`s abdomen*, *I dry and cut the cord*, *if the baby cried it means the clamping is done immediately after birth like within a minute”* SSIP7OB

*“My experience about umbilical cord clamping after birth is within or after 60 seconds*” SSIP5NM

Participant from the focus group discussion reported the different experience:

*“(…)*, *if the baby is well it takes 2–3 minutes and if the baby is not well then you must perform resuscitation first*, *so the time will take a little longer”* FGDP5

#### Sub-theme 2: Clamping the umbilical cord after cessation of cord pulsations

According to WHO delayed cord clamping is done between 1 to 3 minutes following delivery of the baby or after cessation of cord pulsations. Participants in this study reported that they usually wait for the cessation of cord pulsations then they milk the cord towards the baby before they clamp the cord. For example, one participant quoted saying:

*“Before clamping the umbilical cord*, *I consider the cessation of cord pulsations*, *I observe the cord colour*, *and it is advised to pull the blood a little to the baby then followed by clamping”* SSIP5NM

*"(*. . . . .*) It is often advised that no cord pulsations should be felt*, *we should leave the transfusion of blood from placenta to the baby to take place and then clamp the cord"* SSIP1NM

#### Sub-theme 3: Timing of the umbilical cord clamping is determined by the condition of the baby

The condition of the baby at the time of delivery guided the participants on whether to clamp the cord immediately or delay. Participants reported that they usually delay cord clamping if the baby required resuscitation so that the baby could benefit from the oxygenated blood from the placenta. They believed that when the mother takes a deep breath while still connected to her baby via the umbilical cord, it will facilitate the initiation of the baby’s breath due to the oxygen carried in the cord blood.

*"My experience is that when the baby is born with good condition (…)*. *There is no need of wasting time is just injecting oxytocin*, *changing gloves and clamping the cord*. *But if the baby delays to cry that is when you also have to delay*, *so it depends on the condition of the baby”* SSIP2NM

A similar experience was reported during FGD:

*“If the Apgar score is not good*, *(…) I will wait a little bit for the ventilation from the mother to the baby to take place while doing the resuscitation and then I will separate the baby”* FGDP10

*“I look at the baby's condition*, *that's what makes me decide to cut the cord*. *I can cut even within four or five minutes but it depends on how the baby’s condition is”* FGDP2

The practice of maintaining warmth and encouraging bonding was reported to also be an important consideration in the timing of cord clamping. This occurs when the baby is dried thoroughly, positioned skin to skin on the mother’s abdomen and covered with a dry cloth before clamping the umbilical cord. Participants explained that encouraging warmth and bonding not only promotes neonatal adaptation to extra-uterine life but is also an initial intervention for the baby requiring resuscitation.

*“When the baby comes out I put her on the mother's chest*, *dry thoroughly to give her warmth and then put her skin to skin contact with her mother cover the baby well to establish bonding*, *after that*, *I will get into the task of cutting the cord”* SSIP1NM

*“After putting the baby skin to skin contact with her mother*, *I do a quick assessment to exclude any complications*, *I make sure the baby is dry and then I clamp the cord”* SSIP8OBS

### Theme two: Perceptions about the umbilical cord clamping

Early cord clamping is generally carried out within the first 60 seconds of birth and often in the first 15–30 seconds, whereas delayed or late cord clamping generally occurs within 1 to 3 minutes or when the cord pulsations have ceased. This theme describes the two sub-themes generated, that include 1. Perceived benefits of DCC and 2. Perceived risks of DCC. Participants explained the perceived reasons that would make them decide for immediate and delayed cord clamping.

#### Sub-theme 1: Perceived benefits of delayed umbilical cord clamping

Participants reported several benefits of DCC, mainly related to placental transfusion. These were: continuous oxygenation, increased blood volume and haemoglobin level, enhanced extra-uterine respiratory adaptation, the provision of nutrients, and increased immunity. They believed that there is an advantage of getting oxygen to the baby who might be struggling to breathe through the blood carried through the cord. This encouraged some of the providers to delay clamping the cord to prevent hypoxia. One of the participants expressed that:

*“There is something we talk about maternal circulation; the baby may get oxygenation because there is still a mother's blood in the cord so the baby can get oxygenation let us say the RBCs will be still carrying oxygen and the baby gets from the mother”* SSIP8OBS

*“it is possible to clamp immediately when the baby comes out*, *but if there will be problems with breathing to the baby we will still need oxygen from the mother to prevent hypoxia which may be caused by limited oxygen”* FGDP9

*"In my thinking by delaying on cutting the umbilical cord first helps even with the prevention of anaemia through increased haemoglobin level as the baby will continue to get through cord from the placenta"* FGDP4

Early bonding and warmth were also seen as the benefit of delayed cord clamping by informants. They reported that after birth, the skin to skin contact of the baby on the mother’s chest allows thermoregulation, facilitates early initiation of breastfeeding, and promotes bonding. Participants had this to say;

*“(*. . .*) In delayed cord clamping the baby gets oxygen*, *nutrients and immunity”* SSIP9OBS

*"(*. . .*) With delayed clamping*, *there is a bonding made between the mother and the baby*, *as well as the baby gets warmth from the mother through the skin to skin contact that also helps with the stimulation of the baby to become more active"* SSIP6NM

Other participants reported the benefits of nutrients and immunity from delayed cord clamping.

*"There are other substances that are in the placenta and are beneficial to the baby if left longer before clamping which I think the baby will be still getting*, *like nutrients"* FGDP4

#### Sub-theme 2: Perceived risks of delayed umbilical cord clamping

Participants from this study reported blood volume overload, reverse transfusion (neonate to placenta transfusion), hypothermia, and transmission of infection as risks of delayed umbilical cord clamping. One participant had this to say about blood overload;

*“The risks of delayed cord clamping there is something called a transfusion that can cause blood volume overload in the baby`s blood circulation”* SSIP7OBS

Another participant thought that delayed cord clamping may cause reverse transfusion of blood from the baby to the placenta.

*“In my perception (*. . .*)if I continue leaving the baby for so long without clamping the blood may come back from the baby towards the placenta”* SSIP4NM

Transmission of infection through the umbilical cord was also reported to be the concern for early cord clamping:

*“(*. . .*) If we delay on clamping*, *there is a transmission of infection or there may be a high risk of infection transmission from mother to baby”* SSIP7OBS

*“The reason which makes us find ourselves cutting the cord immediately is preventing the transmission of HIV from the mother to the baby (*. . .*) so at birth*, *we cut the cord quickly to prevent that”* FGDP7

However, participants during group discussion raised different concerns about whether or not delayed cord clamping promoted the transmission of infection. Some participants expressed the fact that the risk of infection occurs because of birth trauma and not through the blood carried by the cord. Other participants reported needing a standard to adhere to regarding the recommended time for cord clamping, as described below:

*"I have different ideas for I know that the mode of transmission of HIV from mother to baby is due to lacerations or trauma during childbirth but not through the cord so if it is a standard that the cord clamping if the condition of the baby is good*, *should be after maybe 2 minutes or after 1 minute I think that is a good thing"* FGDP9

*“I do not think there is a transmission of infection from the mother to the baby because of delayed umbilical cord clamping”* FGDP3

#### Theme three: Factors influencing the practice of delayed umbilical cord clamping

In this study, nurse-midwives`and obstetricians`reported personnel and health care facilities factors that may also influence the application of evidence on the timing of umbilical cord clamping. The analysis generated five sub-themes, namely; 1. good knowledge about umbilical cord clamping, 2. using guidelines and Standard Operating Procedures (SOPs) in the labour and delivery room, 3. adequate skilled human resources and medical supplies in the delivery room, 4. regular supportive supervision 5. availability of on-the-job training.

#### Sub-theme 1: Good knowledge about umbilical cord clamping

Having up-to-date knowledge about the risks and benefits of early vs delayed cord clamping would facilitate a change in practice. Study participants explained that this lack of knowledge made them unable to practice delayed cord clamping, as recommended by the current evidence. One of the participants said:

*“I need to have enough knowledge about delivery because every day there is changes concerning delivery”* SSIP2NM

Being open to new ideas was also reported as key to facilitating changes in current practice, as this would enable a willingness to follow new recommendations:

*“(*. . .*) we don’t need anything rather than changing our attitude; so*, *what I mean on changing the attitude is that we have to agree with the recommended time*, *we should not practice in our way we should be ready to change because people have invested in research to give us findings and simplified by showing us which is a safe practice and which is not"* SSIP7OBS

#### Sub-theme 2: Using guidelines and standard operating procedures

In addition to updating knowledge, nurse-midwives`and obstetricians`frequently spoke about the need to use guidelines and standard operating procedures (SOPs) to follow during deliveries. They explained that guidelines on care during delivery could be used to help them provide quality care to women in labour, leading to improved maternal and newborn outcomes. One of the participants expressed that;

*"My thinking is that every facility should have a SOP in addition to that of WHO*, *I know there is a national guideline*, *but standard operating procedures should be available for everyone for example of myself it is not like I don't know things*, *it is possible because there is no SOP that reminds me every time that's why I take things just simple as a routine"* SSIP7OBS

Participants in the FGDs reported similar concerns. They discussed the importance of displaying written protocols in a central location where everyone could read them to stay updated on new practice guidelines:

*“SOPs and protocol are very important (*. . .*)*, *so when SOP is displayed on notice board every nurse-midwife will visit the notice board and read the information that says how long should it take before clamping or separating the baby from the mother"* FGDP10

*“(*. . .*) what can help us more is the availability of standard or guideline which show at what time we should clamp because changes are there and occur often*, *what I remember previously during my studies we practiced the immediate cord clamping but now things have changed that we have to wait for 1 minute before clamping even if the baby`s Apgar score is low”* FGDP9

#### Sub-theme 3: Adequate skilled human resources and medical supplies

Participants pointed out that their high workload due to staff and equipment shortages made it difficult for them to perform some of the current procedures. They explained that working alongside adequately skilled providers could also influence them on how to implement best practices. They reported that they often practiced early cord clamping because of the high number of patients they were responsible for, leading to not having the time to delay cord clamping. One participant expressed that;

*“Sometimes we are overloaded*, *I have to receive the baby put on her mother`s chest*, *cover them well and go to the other client do the same something wrong*, *so adequate number of staffs will help us to clamp the cord at the right time”* SSIP2NM

Because of the difficult working environment due to the shortage of staff and lack of equipment (such as watches that could record the time of delivery and help guide the midwife and/or obstetrician in the timing of cord clamping), providers did not feel able to apply the most current guidelines, even when they were familiar with these guidelines.

*"The things that are required are motivation differently*, *for example*, *improving the working environment*, *ensuring an adequate number of staffs*, *good ratio of providers and clients can help to improve the quality of care provided”* SSIP8OBS

Some providers reported that having adequate equipment, especially for resuscitation, would help them delay clamping the cord, as they would be able to provide resuscitation at the bedside. Sometimes a lack of basic equipment forced them to immediately clamp the cord since they needed to emergently move the newborn to another room where the equipment for resuscitation was available. They reported having only one table set up specifically for newborn resuscitation for all the providers in the labour ward. For this reason, it was not possible to have the equipment available at the bedside.

*“Equipment to help the baby breathe should also be available in the delivery room where it will slow down the speed of cutting the cord quickly for the aim of resuscitation somewhere else*, *but if tools are available there*, *resuscitation can be done on bedside while still there a connection"* SSIP9OBS

#### Sub-theme four: Regular supportive supervision

In this study, participants suggested regular supportive supervision as a strategy that would influence the good practice of delaying umbilical cord clamping. They reported that professional and technical guidance is important to be sure that they are providing the best quality of care. They remarked that supportive supervision with trained providers and specialists could provide updates to their knowledge base and help them to demonstrate best practice. One participant explained that;

*“Any leader may be reminding us through supervision because the way I am doing all things I may not be doing correctly even though I have been taught in class what I am supposed to do but at some point*, *someone else can see what I am doing I should probably make some changes”* SSIP4NM

FGD participants reached a consensus that supervision from experts can help to identify areas in which more training is needed. As one participant pointed out;

*"on my side*, *I see that supervision should be available for people to know that they are required to practice while following guidelines in conducting deliveries*, *we do have regular supervision but in the issue of timing of cord clamping we have never been trained or supervised"* FGDP5

#### Sub-theme 5: Continuous and sustainable on-the-job training

The availability of on-the-job training can increase knowledge levels and improve the clinical skills of the midwives and obstetricians. Providers reported that they had been conducting ongoing medical education every two weeks to improve their clinical practice since they had never been trained on the importance of delayed cord clamping. They explained that more in-service training on delayed cord clamping would encourage them to practice better care for new-borns. One participant had this to say:

*"People should be trained to be aware of the current changes regarding the timing of umbilical cord clamping*, *its benefits and other things about conducting delivery for us to practice as per standard*, *so we should be given that education"* SSIP5NM

Most participants thought that on-the-job training and continuing medical education sessions about the risks and benefits of delayed and immediate cord clamping, respectively, could be very helpful. They expressed the fact that these sessions could help identify areas of practice needing improvement that could then be addressed:

*“Regularly we need to get in-service education sessions to remind us that there are some changes so that we also change our practice because sometimes we tend to overlook some of the areas”* SSIP3NM

*"We should be given education through continuing medical education (CME); we may be given information about changes on a certain practice through on-job training for everyone to be aware of the available changes"* FGDP6

## Discussion

### Experiences about the timing of umbilical cord clamping

In this study, participants employed different practices on the timing of umbilical cord clamping based on their experience during deliveries and their current knowledge base. Those who clamped the umbilical cord within 60 seconds following an uncomplicated birth did not understand the difference in neonatal outcome between early or delayed clamping. These variations in practice are similar to those noted in the literature. Other studies found optimal timing for cord clamping is a challenge with a wide-variation [20, 22], delayed cord clamping being widely-used following uncomplicated vaginal term and preterm deliveries, with the cessation of cord pulsation as the point in time for cord clamping [21, 23]. However, an observational study done in Canada showed that more than half (56.2%) of all infants had their umbilical cords clamped within 15 seconds after a spontaneous vaginal birth [20]. In other countries, delayed umbilical cord clamping has been a standard of care, regardless of the condition of the baby or the possibility of long-term benefits beyond the neonatal period [11, 22, 39].

In the present study, providers who had been clamping the cord after cessation of pulsations had done so because they had noticed positive changes in the neonate`s muscle tone and colour. Others assessed the infant`s condition before clamping to provide initial resuscitation, if needed, and to encourage warmth through skin-to-skin contact. One possible explanation of these practice variations could be that both nurse-midwives and obstetricians were not aware of the WHO and Tanzania National Anaemia Profile recommendations about delayed cord clamping. In this study, the number of years working in maternal and newborn health care did not appear to be associated with any differences in the timing of umbilical cord clamping.

### Perceptions about umbilical cord clamping

The risks and benefits of delayed umbilical cord clamping, as reported by the participants in this study some were inconsistent with the evidence as seen in the literature. This demonstrates the importance of adequately training the providers on matters underlying childbirth. Providers were more likely to practice delayed cord clamping for continuous oxygenation. The reasons they cited for delayed cord clamping were increased blood volume and haemoglobin levels, increased pulmonary blood flow, initiation of extra-uterine respirations, provision of nutrients, and increased immunity. Other potential benefits like lowering the newborn risks which have been reported by the most recent literature were not discussed by participants in this study. Even in high-income countries like the USA, the current literature on the timing of cord clamping is not reflected in the practice [40]. Several studies have revealed the benefits in placental transfusion, increased haematocrit level, cerebral oxygenation and blood pressure, gradual transition to extrauterine, and long-term neurodevelopmental outcomes [5, 9, 41–43].

Furthermore, some risks reported by participants were not substantiated by the current evidence and may need to be addressed through training. Their reasons for not practicing delayed cord clamping were to prevent foetal-placental transfusion, hypothermia, and infection transmission. However, other participants disagreed with these assumptions. According to available evidence, infections such as HIV cannot be transmitted through delayed cord clamping. Moreover, evidence has shown that delayed cord clamping is associated with placental transfusion towards the baby and decreases the risk of blood transfusion [42, 44]. There is no evidence that foetal to placental transfusion occurs, causing anaemia to the neonate. Evidence shows that immediately after the baby is born, placental blood continues to flow in the direction of the infant as long as he or she is positioned up to 10 cm above the level of the placenta [45]. In this study, positive perceptions toward DCC were markedly increased among young professionals and those with fewer years in practice. This is different from what was observed in Saudi Arabia where higher rates of positive attitudes toward DCC were noted among those with more years of obstetrics and gynaecology practice [22].

### Factors influencing the practice of delayed umbilical cord clamping

This study revealed that the implementation of DCC would be influenced by increased knowledge about the benefits of umbilical cord clamping, the use of guidelines and SOPs, as well as more resources in the areas of clinical training, and equipment (needed for resuscitation). It was noted that the timing of umbilical cord clamping can be improved if providers are given up to date knowledge and skills through on-the-job training. This is illustrated by higher-income countries where most providers practiced delayed cord clamping due to having access to up to date information about the risks and benefits of delayed cord clamping through clinical training [16, 21, 24].

The need for guidelines and SOPs for providers on the optimal timing of umbilical cord clamping was also shown in this study. Participants acknowledged the existence of the WHO guidelines, but they weren’t sure about any National Guidelines which focused on the timing of umbilical cord clamping. This was also reported in other countries where there was a wide variation of umbilical cord clamping practices [21–24, 30, 31]. The availability of guidelines could be beneficial in educating and providing guidance to providers [46]. A shortage of resources was reported to be a possible factor for not applying the evidence of umbilical cord clamping. Due to a lack of providers in labour and delivery, the staff were overwhelmed with work. This contributed to early clamping of the umbilical cord, particularly when more than one woman was in the pushing stage of labour and there was only one midwife available. More staff and equipment could potentially increase the practice of delayed cord clamping.

Since most participants were unaware of the potential benefits of delayed cord clamping, more in-service training, and clinical supervision were suggested as ways to improve their practice. Regular continuing medical education sessions would also increase the provider's knowledge level and skills [21, 24]. This study demonstrated that there was a great need for in-service training not only to update current knowledge but also as a way to remind providers about the importance of maintaining their clinical skills. Interdisciplinary training sessions could also provide a forum for providers to discuss any concerns about the practice of umbilical cord clamping. This was demonstrated in Saudi Arabia where there was a need for educational programs on the benefits of delayed cord clamping for midwives to improve their knowledge and practice [22].

It is well documented that regular clinical supervision of providers is an efficient way to convey updates on clinical practice, such as the implementation of delayed cord clamping [24]. In this study, participants reported regular monitoring and supportive supervision from the Ministry of Health, the Regional Health Management Team (RHMT), and Non-Governmental Organizations working in the municipality. However, this supervision never included the practice of umbilical cord clamping. Successful implementation of the current guidelines about the optimal timing of umbilical cord clamping would depend on regular supportive supervision that focuses on this particular area of practice [24].

### Methodological considerations

The researcher ensured credibility through the use of multiple data sources (nurse-midwives`and obstetricians`) and through combining data collection methods (semi-structured interviews and focus group discussion) which allowed the triangulation of results. Triangulation of data sources and methods of data collection ensured authenticity and comprehensive understanding of the phenomenon thus increased validity and reliability of the findings [36, 47, 48]. The use of the Kiswahili language also enhanced the credibility of the findings, given the fact that Kiswahili is the National language and spoken comfortably by all participants. The use of Kiswahili language during data collection was important because the qualitative study works with words, therefore it was critical to ensure that concepts are understood and limit the risk of misinterpretation. It also ensured that participants’ accounts are not distorted by translation during the analysis process [49].

The interviews were conducted by the first author (DLM), a midwife working at the University. Conducting interviews with the nurse-midwives may introduce some bias. Participants may over or under-report their experiences. To reduce this bias, the researcher applied various interviewing techniques including probing the same questions in different ways and being naïve on the topic during the interview process [50]. Further, as one of the midwife specialists with an adequate understanding of the reason to apply delayed cord clamping, it is likely that in some ways she has influenced the interpretation of data. Nevertheless, data analysis was done jointly with the second author (LTM) and the generated themes were dialogue and collective decision on the naming of the themes was reached.

## Conclusion

Results from this study demonstrate a variety of practices by a cohort of nurse-midwives`and obstetricians`on the timing of umbilical cord clamping following delivery. Despite the nurse-midwives’ and obstetricians’ experience of clamping the umbilical cord immediately or within 60 seconds, they acknowledged that delayed cord clamping has the potential benefit of oxygenation to the newborn requiring resuscitation. Perceived risks of delayed cord clamping, such as the transmission of infection, though not substantiated by current evidence, were reported by the majority of study participants. This calls for proper training of the providers on matters underlying childbirth to address this gap of knowledge.

Nurse-midwives and obstetricians should be encouraged to practice based on evidence. Therefore, the availability of guidelines including SOPs to support practice has been seen to be more important in influencing good practice. Further, the management of regional hospitals should strive to improve the working environment for the national health policy on access to maternity care to be realized. Moreover, the findings call for the Ministry of Health and other stakeholders to review and adopt the existing guidelines and protocols to ensure that DCC is considered at every delivery as it has shown a marked impact on newborn outcomes.
